# *THY1* is a prognostic-related biomarker via mediating immune infiltration in lung squamous cell carcinoma (LUSC)

**DOI:** 10.18632/aging.205880

**Published:** 2024-05-30

**Authors:** Changsheng Yi, Nan Zang, Limin Gao, Fang Ren

**Affiliations:** 1 The Affiliated Cancer Hospital of Zhengzhou University and Henan Cancer Hospital, Zhengzhou 450008, China; 2 Henan Provincial Chest Hospital, Zhengzhou University, Zhengzhou 450000, China; 3 Department of Obstetrics and Gynecology, First Affiliated Hospital, Shihezi University, Shihezi, Xinjiang 832000, China; 4 Department of Obstetrics and Gynecology, First Affiliated Hospital of Zhengzhou University, Zhengzhou, Henan, China

**Keywords:** *THY1*, LUSC, immune infiltration, biomarker, bioinformatics

## Abstract

Thymus cell antigen 1 (*THY1*) has been proven to play pivotal roles in many diseases. However, we do not fully understand its functional mechanism, especially in lung squamous cell carcinoma (LUSC). Here, we aimed to perform a comprehensive analysis to explore the expression and prognostic values of *THY1* in LUSC using bioinformatic technology. Some online public databases (e.g., ONCOMINE, PrognoScan, TIMER, Kaplan-Meier plotter, STRING, LinkedOmics, and GEPIA) were used to explore the expression, prognostic significance, and potential molecular mechanism of *THY1*. The analysis indicated that *THY1* was significantly up-regulated and closely correlated with poor prognosis in many malignant tumors, including LUSC. Further analysis revealed that over-expression of *THY1* was significantly correlated with clinicopathological parameters (e.g., individual cancer stage, age, smoking habits, nodal metastasis status, and *TP53* mutation status) in LUSC. The CpG islands methylation of *THY1* was negatively correlated with *THY1* mRNA expression in The Cancer Genome Atlas Program (TCGA). Further enrichment analysis of *THY1* correlated genes revealed that they were mainly correlated with the formation of extracellular matrix (ECM), and got involved in the pathway of epithelial mesenchymal transition (EMT). Furthermore, differentially expressed *THY1* was significantly correlated with immune cell infiltrations and poor prognosis in LUSC. In summary, bioinformatic analysis demonstrated that *THY1* was significantly over-expressed and closely correlated with unfavorable prognosis in LUSC, which may apply as a promising diagnostic and therapeutic biomarker for LUSC in the future.

## INTRODUCTION

Lung cancer (LUCA) is one of the most common malignant diseases around the world, which results in the predominant cancer deaths [1, 2]. It was estimated that there were 116,300 and 112,520 new LUCA and bronchus cancer cases among males and females in the United States by 2020, respectively [3]. The estimated deaths of them were 72,500 and 63,220, respectively. LUCA is mainly composed of small cell lung carcinoma (15–20%) and non-small cell lung carcinoma (NSCLC) (80–85%), and LUSC is a pathological subtype of NSCLC, which accounts for approximately 40% of all LUCA [4, 5]. In recent years, although great progress has been made in the diagnosis and treatments for LUSC, the prognosis of LUSC patients was still unfavorable due to the lack of specific targets and effective targeted drugs compared to lung adenocarcinoma [6, 7]. The local recurrence and distant metastasis (e.g., brain, liver) were common in LUSC, even in early stage. Therefore, it is of great significance to identify more efficient and specific biomarkers for LUSC to prolong patients’ survival.

Thymus cell antigen 1 (*THY1*), also called as cluster of differentiation (CD90). *THY1* is one of cell surface glycoproteins with a molecular weight of 25-37KDa. It is widely expressed in various parts of humans and mice (fibroblasts, neurons, murine T cells, etc.) [8, 9]. It is a key molecule of the interactions between cell and cell or cell and matrix [10]. *THY1* was reported to play pivotal roles in a variety of malignant diseases, and the functions of *THY1* had tissue heterogeneity, which indicated that it could be an oncogene or a tumor suppressor gene at different diseases. For example, *THY1* was over-expressed in undifferentiated hepatocellular carcinoma, and was significantly correlated with poorer prognosis [11]. *THY1* has also been confirmed to be highly expressed in prostate cancer and male breast cancer [12]. Nevertheless, *THY1* played an opposite role in ovarian cancer [13, 14]. At present, there is still a wide gap in our understanding of how *THY1* plays a role in LUSC.

The identification of novel biomarkers can provide a new perspective for the development of targeted drugs and the early diagnosis. Here, we aimed to explore the expression, prognostic significance, and functional mechanism of *THY1* in LUSC using bioinformatics technology.

## RESULTS

### Over-expression of *THY1* in many malignant tumors

**Figure 1:**
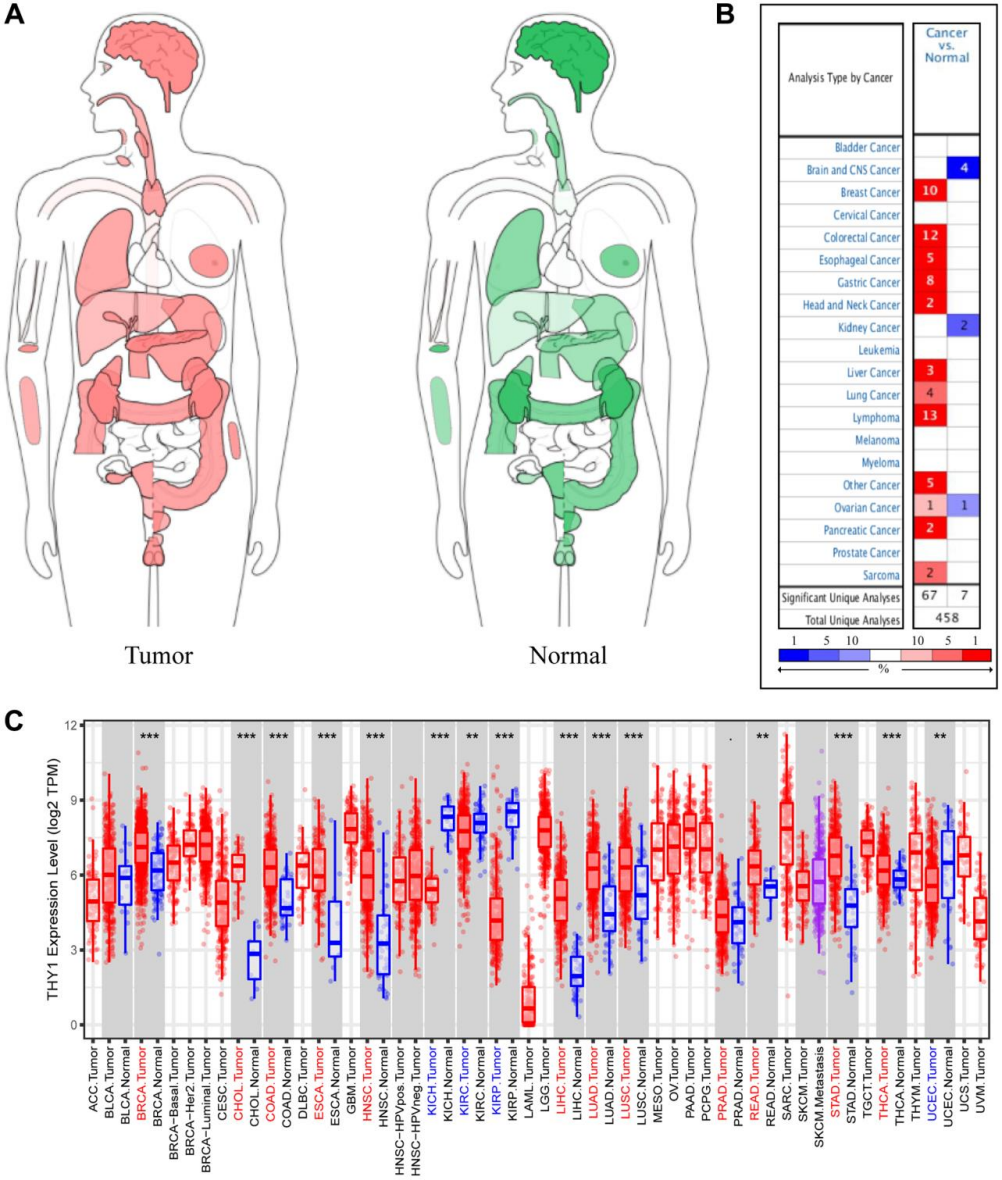
**Differentially expressed *THY1* in a variety of cancers and normal tissues.** (**A**) The interactive body-map revealed the median expression of *THY1* in tumor (red) and normal samples (green) using GEPIA (scale: Log2 (TPM+1)). (**B**) The expression levels of *THY1* in different tumors compared to normal tissues from ONCOMINE database. (**C**) The expression levels of *THY1* in 33 types of cancers compared to normal tissues from TCGA in TIMER database.

We firstly used the GEPIA to explore the expression of *THY1* in tumors and normal tissues. As shown in Figure 1A, *THY1* was widely expressed in a variety of malignant tumors (i.e., brain cancer, lung cancer, colon cancer) and normal organs (i.e., brain, kidney, breast). We further used the ONCOMINE to explore the full landscape of *THY1* expression in different malignant tumors compared to normal tissues. As shown in Figure 1B, *THY1* was significantly up-regulated in many human cancer (e.g., breast cancer, colorectal cancer, liver cancer, gastric cancer, lung cancer), and the down-regulated *THY1* was occurred in brain and CNS cancer, kidney cancer, and ovarian cancer. We also evaluated the expression level of *THY1* in many malignancies in TCGA using TIMER. Results indicated that *THY1* was significantly upregulated in breast cancer, cholangiocarcinoma (CHOL), colon adenocarcinoma, esophageal carcinoma, head and neck squamous cell carcinoma, liver hepatocellular carcinoma, lung adenocarcinoma, LUSC, prostate adenocarcinoma, rectum adenocarcinoma, stomach adenocarcinoma, and thyroid carcinoma. Nevertheless, it was down-regulated in kidney chromophobe, kidney renal cell carcinoma, kidney renal papillary cell carcinoma, and uterine corpus endometrial carcinoma (Figure 1C).

### The prognostic significance of differentially expressed *THY1* in many cancers

**Figure 2:**
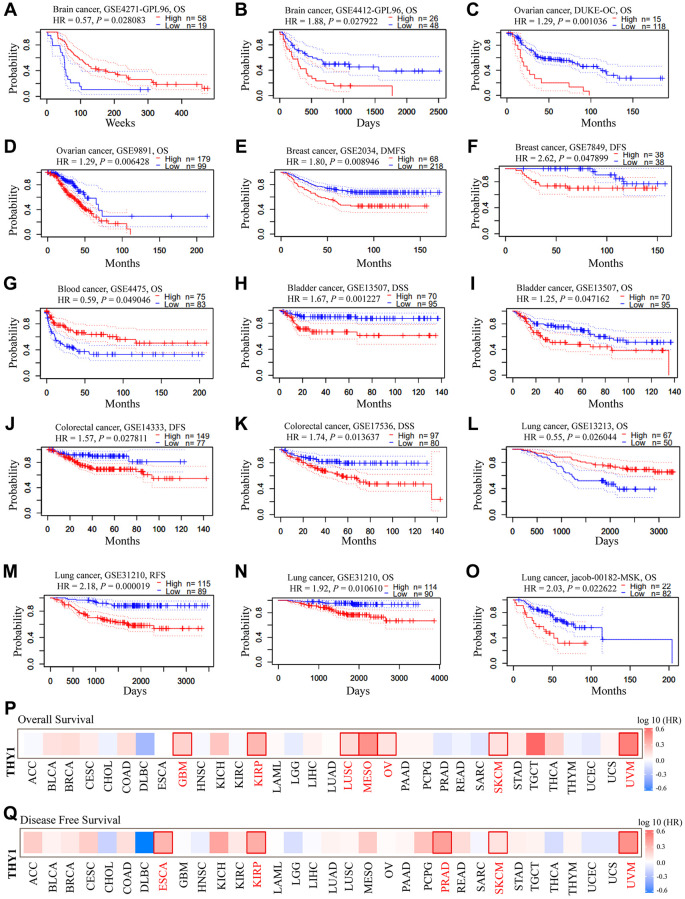
**The prognostic significance of up-regulated *THY1* in many types of cancers.** (**A**–**O**) Relationships between higher expression of *THY1* and prognosis in different types of cancers in PrognoScan database. (**P**) The prognostic value (OS) of differentially expressed THY1 in 33 types of cancer form TCGA in GEPIA database. (**Q**) The prognostic value (DFS) of differentially expressed THY1 in 33 types of cancer from TCGA in GEPIA database. Abbreviations: OS: overall survival; DFS: disease-free survival; DMFS: disease-metastasis free survival; DSS: disease specific survival; RFS: relapse free survival.

We then explored the prognostic significance of *THY1* in LUSC using the PrognoScan database. As shown in Figure 2A–2O, up-regulated *THY1* was significantly correlated with the poorer prognosis in many malignancies. However, over-expression of *THY1* also favored a better prognosis in some cancers. We further used the GEPIA to measure the prognostic values of *THY1* in 33 types of cancers from TCGA. As shown in Figure 2P and Supplementary Figure 1, elevated *THY1* significantly correlated with poor overall survival (OS) in glioblastoma multiforme, kidney renal papillary cell carcinoma, LUSC, mesothelioma, ovarian cancer, skin cutaneous melanoma, and uveal melanoma. Meanwhile, over-expression of *THY1* also predicted poor (DFS) in esophageal carcinoma, kidney renal papillary cell carcinoma, prostate adenocarcinoma, skin cutaneous melanoma, and uveal melanoma (Figure 2Q and Supplementary Figure 2). Results from the PrognoScan and GEPIA consistently demonstrated that up-regulated *THY1* significantly predicted poorer survival in many malignant tumors, including LUSC.

### The relationships between *THY1* expression and clinicopathological parameters in LUSC

**Figure 3:**
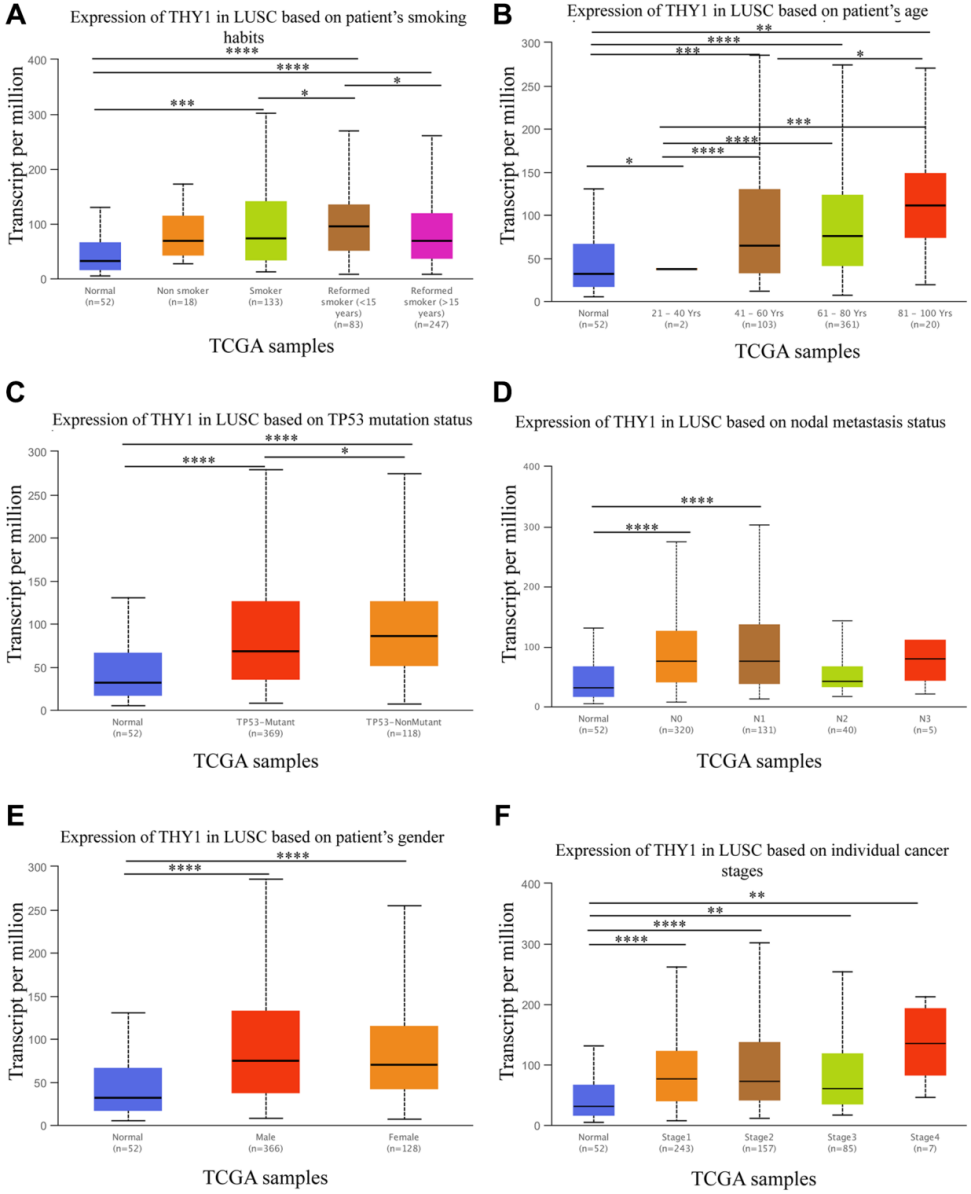
**Relationships between *THY1* expression and clinicopathological parameters in LUSC.** (**A**–**F**) Relationships between *THY1* expression and patient’s smoking habits, age, TP53 mutation status, nodal metastasis status, gender, and individual cancer stage in LUSC from UALCAN database. ^*^P < 0.05; ^**^P < 0.01; ^***^P < 0.001; ^****^P < 0.0001.

Based on the above results, we found that *THY1* was significantly up-regulated and correlated with poor prognosis in LUSC. We then further explored the relationships between *THY1* expression level and clinicopathological parameters in LUSC using the UALCAN. As shown in Figure 3A–3F, *THY1* was significantly correlated with smoking habits, age, TP53 mutation status, nodal metastasis status, gender, and individual cancer stages in LUSC. We also measured the relationships between *THY1* expression and prognosis in LUSC under different clinicopathological parameters using the Kapan-Meier plotter. However, there is little correlation between *THY1* expression and OS, first progression (FP), and post progression survival (PPS) under these restricted situations (Supplementary Table 1).

### The methylation level of *THY1* in LUSC and normal tissues

**Figure 4:**
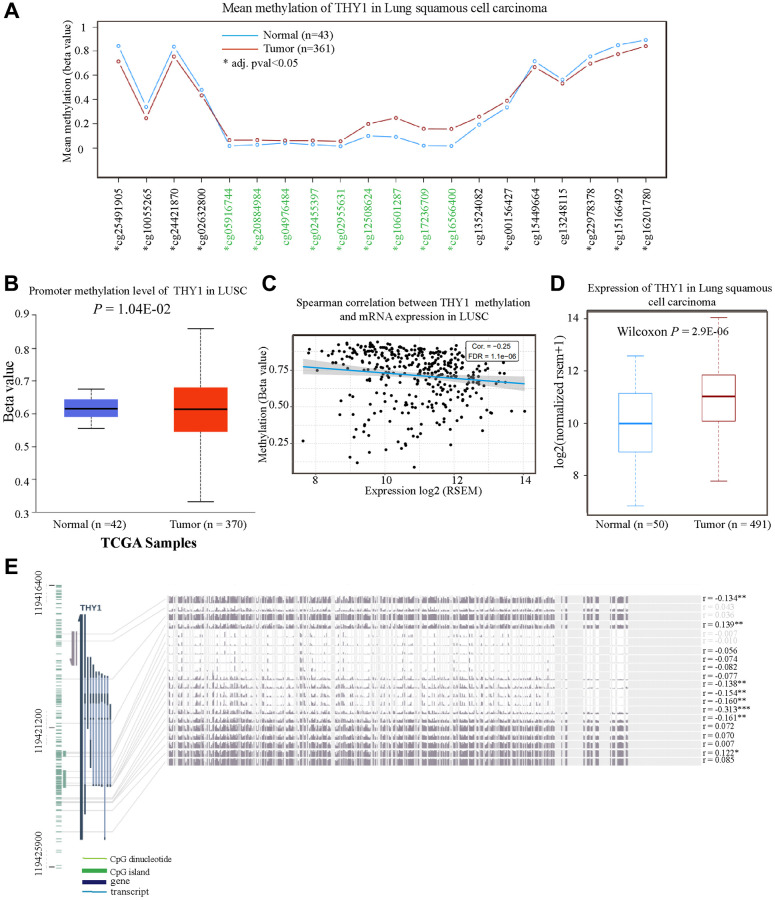
**The methylation level of *THY1* in LUSC and normal tissues.** (**A**) Mean methylation level of *THY1* in LUSC and normal tissues regarding different probes using the TCGA Wander. (**B**) Promoter methylation level of *THY1* in LUSC using UALCAN. (**C**) Relationships between *THY1* methylation and mRNA expression in LUSC using GSCA. (**D**) Expression level of *THY1* in LUSC and normal tissues using TCGA Wander. (**E**) Relationships between different *THY1* CpG islands methylation and *THY1* expression using MEXPRESS. ^*^P < 0.05; ^**^P < 0.01; ^***^P < 0.001.

Many studies have confirmed that the dysregulation of oncogenes or tumor suppressor genes DNA methylation played pivotal roles in the occurrence and development of malignant tumors [15]. Here, we used some online databases to explore the methylation level of *THY1* in LUSC and normal tissues. As shown in Figure 4A, 8 probes (8/20) indicated the methylation of *THY1* in LUSC were lower than that in normal tissues using the TCGA Wander. The average promoter methylation of *THY1* in LUSC was also lower compared to normal tissues (*P* = 1.041E-02) using the UALCAN (Figure 4B). Furthermore, the *THY1* methylation level was negatively correlated with its mRNA expression (Cor = −0.25, FDR = 1.1E-06), and the *THY1* was also significantly up-regulated in LUSC compared to normal tissues (Figure 4C, 4D). Finally, we also used the MEXPRESS to measure the relationships between different *THY1* CpG islands methylation and *THY1* expression level. As shown in Figure 4E, we identified 6 CpG islands among 16 CpG islands of *THY1* that were significantly correlated with *THY1* expression. In summary, these results consistently indicated that CpG islands methylation of *THY1* played pivotal roles in over-expression of *THY1* in LUSC.

### Functional enrichment analysis of identified differentially expressed genes correlated with *THY1* in LUSC

**Figure 5:**
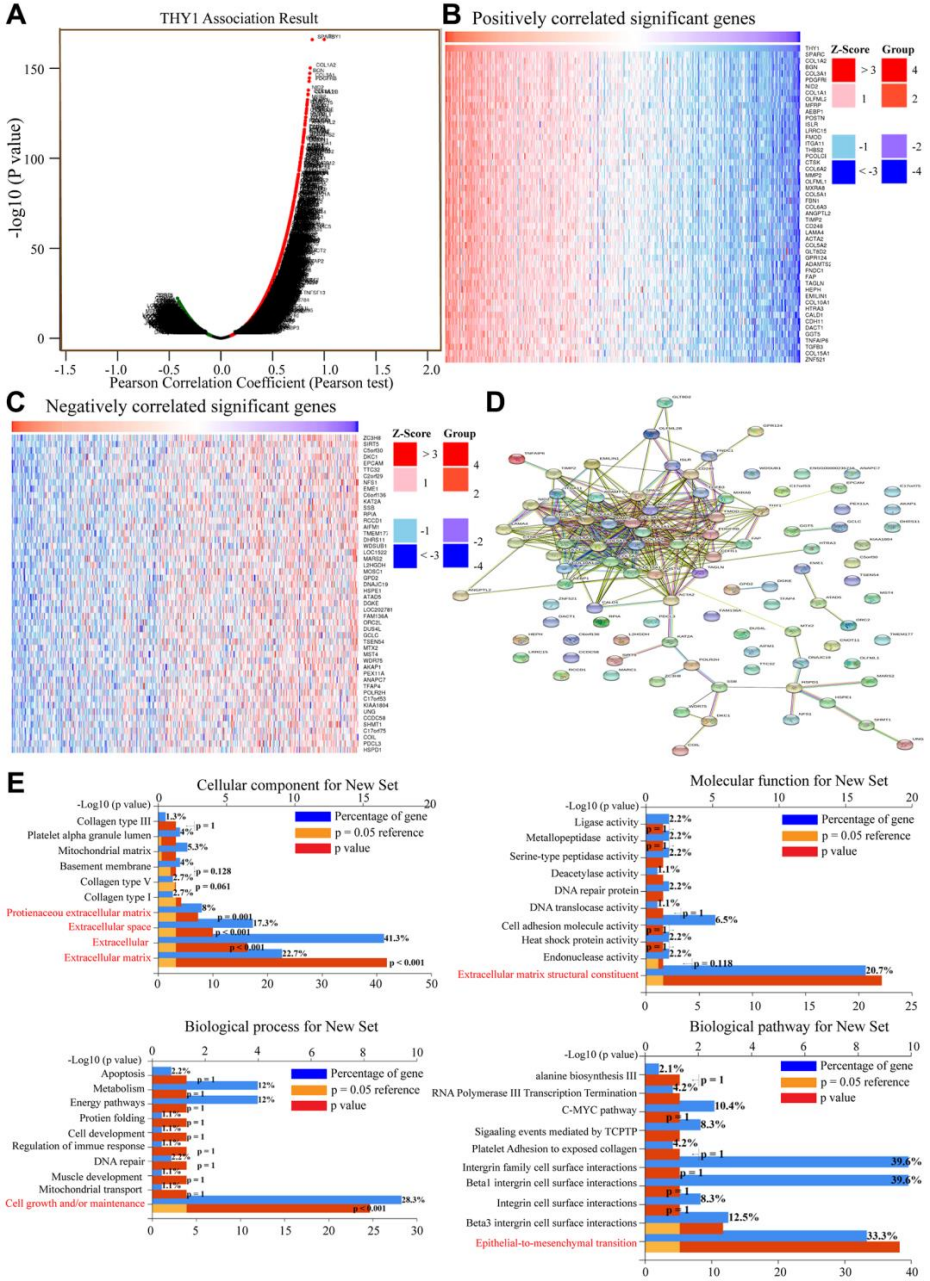
**Functional enrichment analysis of correlated genes of *THY1* in LUSC.** (**A**) Genes that positively or negatively correlated with *THY1* in LUSC measured by Pearson test in LinkedOmics. (**B**) Heatmap of positively correlated genes of *THY1* in LUSC. (**C**) Heatmap of negatively correlated genes of *THY1* in LUSC. (**D**) Protein-protein interactive network of top correlated genes constructed by STRING. (**E**) Functional enrichment analysis of these top correlated genes regarding cellular component, molecular function, biological process, and biological pathway using FunRich 3.v.13.

We also aimed to further explore the potential mechanism mediated by *THY1* in LUSC. We identified genes that were positively or negatively correlated with *THY1* in LUSC using the LinkedOmics. As shown in Figure 5A, there were 10,495 positively correlated and 9,608 negatively correlated genes of *THY1* in LUSC *(P <* 0.05). The top 50 positively and negatively correlated genes were displayed in Figure 5B, 5C. The protein- protein interaction network (PPI) network of these top genes was constructed using the STRING (Figure 5D). Functional enrichment analysis of these 100 correlated genes indicated that they were mainly correlated with the formation of ECM, and were got mainly involved in the process of EMT (Figure 5E).

### *THY1* expression is correlated with immune cell abundance in LUSC

**Figure 6:**
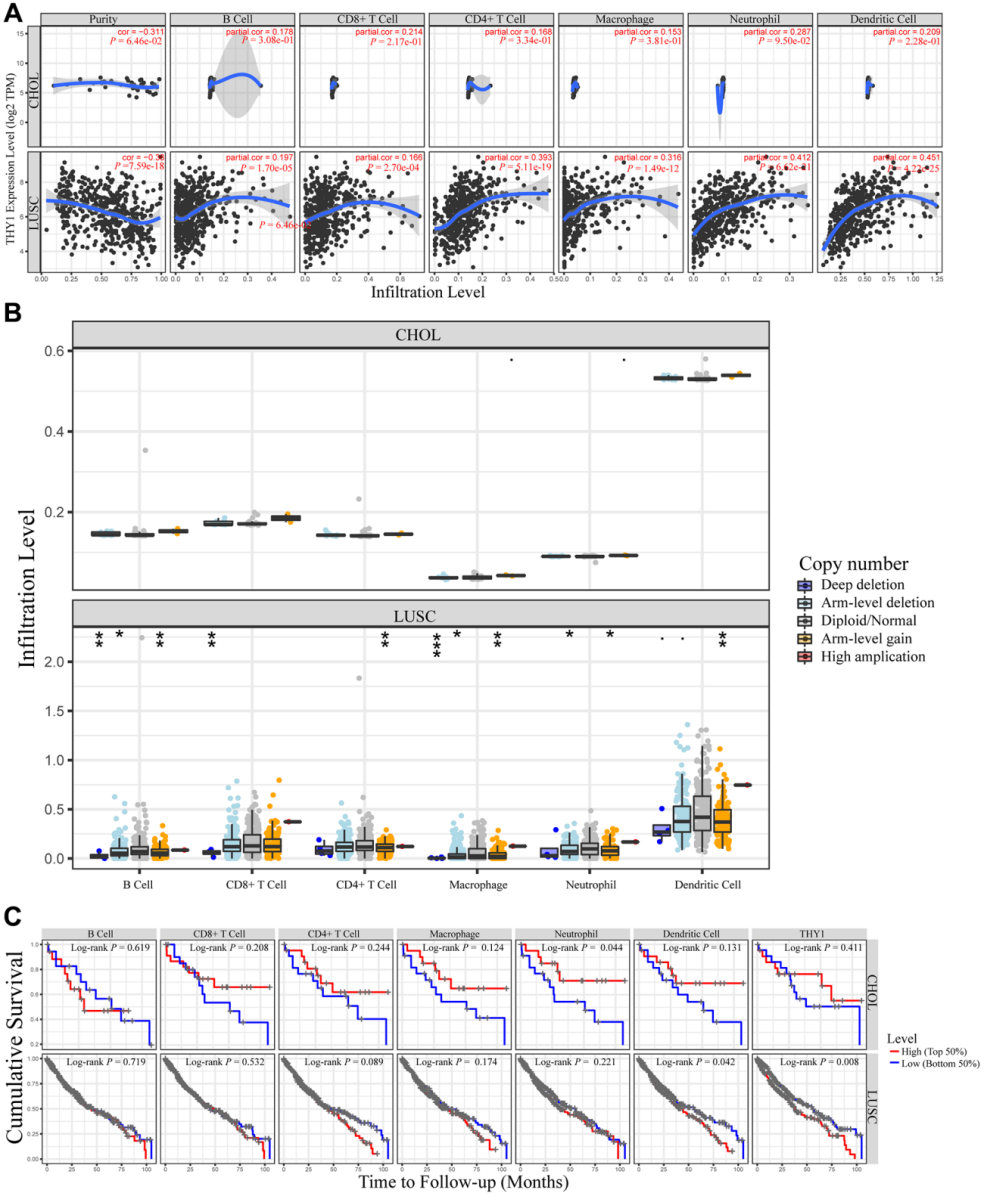
**Relationships between *THY1* expression and immune infiltration levels in lung squamous cell carcinoma (LUSC) and cholangiocarcinoma (CHOL).** (**A**) Differentially expressed *THY1* was significantly correlated with lymphocytes infiltration levels in LUSC, but not in CHOL. (**B**) Copy number alteration of different immune lymphocytes was significantly correlated with immune infiltration levels in LUSC, but not in CHOL. (**C**) Higher *THY1* expression and dendritic cell infiltration level significantly correlated with poorer prognosis in LUSC.

Studies had indicated that the tumor immune microenvironment was closely related to the prognosis of patients, and the abundance of tumor infiltrating immunocytes played a pivotal role [16, 17]. Hence, we further measured the relationships between *THY1* expression and immune cell infiltrates in 39 types of cancer from TCGA. As shown in Figure 6A and Supplementary Figure 3, there was a significant correlation between *THY1* expression and tumor purity in 26 types of cancers. Among them, *THY1* expression was negatively correlated with tumor purity (cor = −0.38, *P* = 7.59E-28), and *THY1* expression had a positive correlation with B cell (cor = 0.197, *P* = 1.70E-05), CD8^+^ cell (cor = 0.166, *P* = 2.70E-04), CD4^+^ cell (cor = 0.393, *P* = 5.11E-19), Macrophage (cor = 0.316, *P* = 1.49E-12), Neutrophil (cor = 0.412, *P* = 6.62E-21), and Dendritic cell (cor = 0.451, *P* = 4.22E-15) in LUSC. Nevertheless, there was no significant correlation between *THY1* expression and tumor purity (cor = −0.311, *P* = 6.46e-02) and other immune lymphocytes (*P* > 0.05) in CHOL. CHOL was used as the control in the following analysis.

We then further used the SCNA module of TIMER to explore the tumor infiltration levels under different somatic copy number alterations. As shown in Figure 6B, there were significant correlations between copy number alterations and tumor infiltration levels in LUSC. For example, there were significant correlations between infiltration levels and deep deletion, arm-level deletion, and arm level gain in B cell. However, there were no the same relationships in CHOL.

Meanwhile, the survival module of TIMER was used to measure the relationships between inflammatory infiltration and prognostic value and *THY1* expression in LUSC. Although there were significant correlations between *THY1* expression and the abundance of many immune lymphocytes, only higher dendritic cell infiltration (Log-rank *P* = 0.042) and *THY1* expression (Log-rank *P* = 0.008) significantly correlated with shorter cumulative survival in *THY1* in LUSC (Figure 6C). In summary, *THY1* may play pivotal roles via mediating immune lymphocytes infiltration in LUSC, especially dendritic cell.

### *THY1* expression is significantly correlated with immune marker expression in LUSC

**Figure 7:**
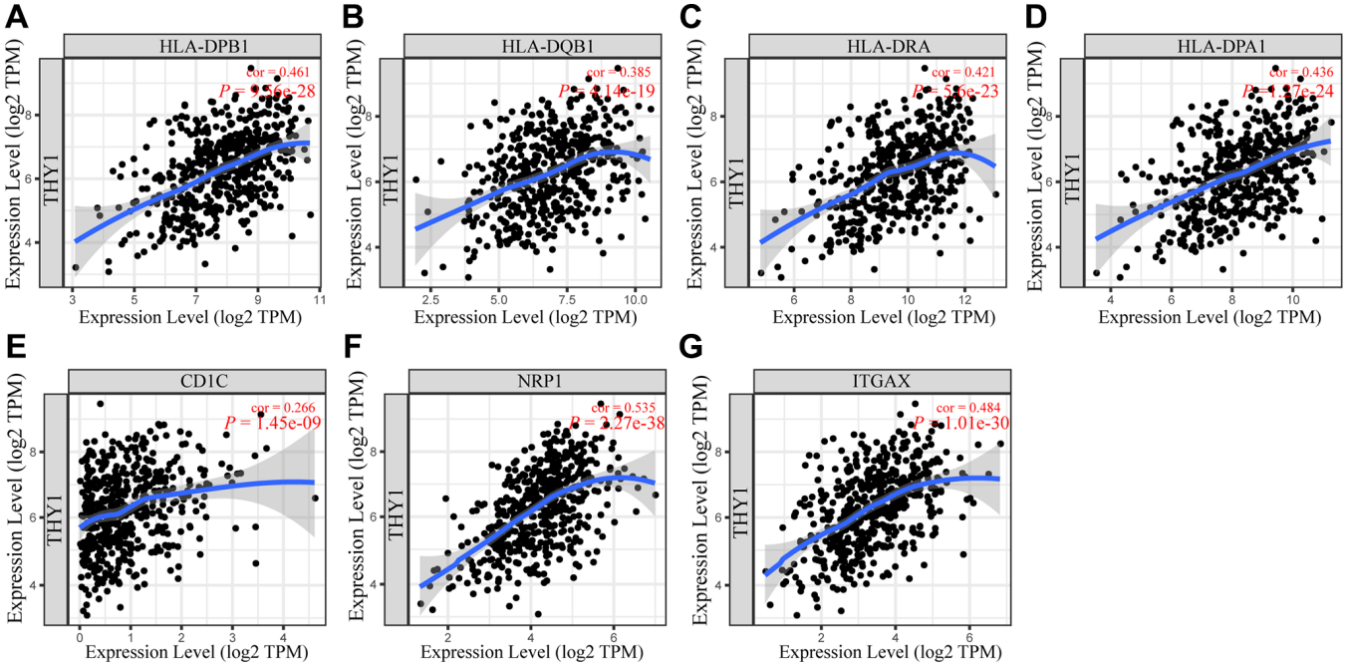
**Associations between *THY1* expression and gene markers of dendritic cells.** (**A**–**G**) The relationships between *THY1* expression and HLA-DPB1, HLA-DQB1, HLA-DRA, HLA-DPA1, CD1C, NRP1, and ITGAX in LUSC from TIMER database.

Next, we explored the relationships between the gene markers of immune lymphocytes and *THY1* expression in order to further validate the function of differentially expressed *THY1* on immunocytes infiltration levels in LUSC. The CHOL was used as the control, and the tumor purity was used to adjust these results. As shown in Table 1, *THY1* was positively correlated with the most of the gene markers, which was consistent with the results of the previous results in LUSC. All these gene markers of dendritic cell (HLA-DPB1, HLA-DQB1, HLA-DRA, HLA-DPA1, BDCA-1, BDCA-4, and CD1c) were significantly correlated with *THY1* expression in LUSC (Figure 7A–7G). However, *NOS2* (M1 macrophages), *IRF5* (M1 macrophages), *CD66b* (Neutrophils), *KIR2DL4* (Natural killer cell), *KIR3DL3* (Natural killer cell), *STAT6* (TH2), and *IL17A* (TH17) were not significantly correlated with *THY1* expression in LUSC, and *BCL6* (Tfh) was negatively correlated with *THY1* expression. On the contrary, there were few significant correlations between *THY1* expression and lymphocytes gene markers in CHOL. Only *NOS2* (M1 macrophages), *CD163* (M2 macrophages), *MS4A4A* (M2 macrophages), *CD66b* (Neutrophils), *TBX21* (Th1), *TGFBA* (Treg) were positively correlated with *THY1* expression. Only CD66b of neutrophil gene markers were positively correlated with *THY1* expression in CHOL (cor = 0.352, *P* < 0.05), which was not completely consistent with the previous prognostic analysis of neutrophil in CHOL. Finally, we further used GEPIA to explore the relationships between *THY1* expression and the gene markers of dendritic cell gene in LUSC. As shown in Table 2, *THY1* expression was positively correlated with these gene markers in LUSC. In summary, these results strongly proved that *THY1* may play a key role in LUSC by regulating the infiltration of immune cells, especially for the infiltration abundance of dendritic cells.

**Table 1:** Correlation between THY1 and gene markers of immune infiltrates in LUSC and CHOL from TIMER database.

**Description**	**Gene markers**	**LUSC**				**CHOL**			
		**None**		**Purity**		**None**		**Purity**	
		**Cor**	***P***	**Cor**	***P***	**Cor**	***P***	**Cor**	***P***
CD8^+^ T cells	CD8A	0.271	^****^	0.17	^***^	0.204	0.231	0.077	0.661
	CD8B	0.22	^****^	0.169	^***^	−0.082	0.633	−0.213	0.22
T cell (general)	CD3D	0.355	^****^	0.23	^****^	−0.368	0.0278	0.255	0.14
	CD3E	0.43	^****^	0.312	^****^	0.274	0.105	0.119	0.497
	CD2	0.393	^****^	0.273	^****^	0.229	0.179	0.069	0.695
B cell	CD19	0.34	^****^	0.189	^****^	0.278	0.1	0.147	0.398
	CD79A	0.425	^****^	0.295	^****^	0.285	0.0926	0.162	0.352
Monocyte	CD86	0.472	^****^	0.36	^****^	0.233	0.171	0.083	0.634
	CD115 (CSF1R)	0.575	^****^	0.488	^****^	0.116	0.5	−0.012	0.945
TAM	CCL2	0.561	^****^	0.504	^****^	0.285	0.092	0.23	0.184
	CD68	0.409	^****^	0.3	^****^	0.09	0.6	0.003	0.989
	IL10	0.445	^****^	0.379	^****^	0.283	0.0941	0.104	0.551
M1 macrophage	INOS (NOS2)	−0.016	0.723	−0.006	0.899	0.335	^*^	0.35	^*^
	IRF5	0.071	0.111	0.03	0.515	0.007	0.97	−0.074	0.673
	COX2 (PTGS2)	0.222	^****^	0.191	^****^	0.322	0.0562	0.233	0.177
M2 macrophage	CD163	0.461	^****^	0.373	^****^	0.43	^*^	0.335	^*^
	VSIG4	0.401	^****^	0.302	^****^	0.321	0.0566	0.215	0.215
	MS4A4A	0.424	^****^	0.321	^****^	0.452	^*^	0.348	^*^
Neutrophils	CD66b (CEACAM8)	0.081	0.0698	0.054	0.237	0.352	^*^	0.363	^*^
	CD11b (ITGAM)	0.523	^****^	0.434	^****^	0.165	0.335	0.095	0.587
	CCR7	0.385	^****^	0.26	^****^	0.212	0.213	0.043	0.808
Natural killer cell	KIR2DL1	0.108	^*^	0.052	0.259	−0.001	0.995	−0.05	0.777
	KIR2DL3	0.091	^*^	0.031	0.495	−0.042	0.81	−0.083	0.636
	KIR2DL4	0.045	0.32	-0.042	0.363	−0.117	0.497	−0.202	0.245
	KIR3DL1	0.182	^****^	0.108	^*^	0.018	0.918	−0.029	0.869
	KIR3DL2	0.117	^*^	0.029	0.531	−0.113	0.512	−0.128	0.462
	KIR3DL3	0.029	0.518	-0.049	0.29	−0.083	0.632	−0.133	0.446
	KIR2DS4	0.126	^**^	0.071	0.121	−0.034	0.384	0.015	0.932
Dendritic cell	HLA-DPB1	0.461	^****^	0.349	^****^	0.197	0.249	0.066	0.706
	HLA-DQB1	0.385	^****^	0.295	^****^	0.06	0.725	−0.032	0.854
	HLA-DRA	0.421	^****^	0.31	^****^	0.119	0.487	−0.04	0.82
	HLA-DPA1	0.436	^****^	0.329	^****^	0.134	0.433	−0.019	0.913
	BDCA-1 (CD1C)	0.266	^****^	0.082	0.074	0.195	0.255	0.065	0.712
	BDCA-4 (NRP1)	0.535	^****^	0.466	^****^	0.314	0.0628	0.235	0.174
	CD11c (ITGAX)	0.484	^****^	0.357	^****^	0.168	0.327	0.012	0.947
Th1	T-bet (TBX21)	0.327	^****^	0.21	^****^	0.338	^*^	0.199	0.251
	STAT4	0.429	^****^	0.32	^****^	0.208	0.223	0.125	0.476
	STAT1	0.189	^****^	0.125	^**^	0.118	0.493	0.069	0.693
	IFN-y (IFNG)	0.113	^*^	0.038	0.408	0.12	0.485	−0.031	0.861
	TNF-a (TNF)	0.383	^****^	0.296	^****^	0.205	0.229	0.161	0.356
TH2	GATA3	0.399	^****^	0.339	^****^	0.313	0.0633	0.177	0.309
	STAT6	0.024	0.588	0.024	0.603	0.224	0.189	0.247	0.152
	STAT5A	0.411	^****^	0.317	^****^	0.232	0.173	0.185	0.288
	IL13	0.089	^*^	0.026	0.57	−0.026	0.878	−0.113	0.519
Tfh	BCL6	−0.142	^**^	−0.114	^*^	0.266	0.116	0.254	0.141
	IL21	0.124	^**^	0.045	0.327	0.099	0.567	0.013	0.94
Th17	STAT3	0.261	^****^	0.254	^****^	0.153	0.371	0.158	0.366
	IL17A	0.017	0.699	-0.048	0.295	0.106	0.539	0.033	0.849
Treg	FOXP3	0.52	^****^	0.602	^****^	0.224	0.188	0.075	0.667
	CCR8	0.553	^****^	0.466	^****^	0.151	0.38	0.028	0.873
	STAT5B	0.153	^***^	0.164	^***^	0.247	0.147	0.225	0.193
	TGFB (TGFB1)	0.451	^****^	0.389	^****^	0.501	^**^	0.451	^*^
T-cell exhaustion	PD-1 (PDCD1)	0.33	^****^	0.216	^****^	0.232	0.173	0.16	0.358
	CTLA4	0.442	^****^	0.326	^****^	0.092	0.594	−0.012	0.945
	LAG3	0.264	^****^	0.179	^****^	0.087	0.613	−0.033	0.85
	TIM-3 (HAVCR2)	0.454	^****^	0.347	^****^	0.197	0.249	0.061	0.728
	GZMB	0.236	^****^	0.113	^*^	0.143	0.404	0.006	0.971

Abbreviations: LUSC: Lung Squamous Cell carcinoma; CHOL: Cholangiocarcinoma; TAM: Tumor-associated Macrophage. ^*^P < 0.05; ^**^P < 0.01; ^***^P < 0.001; ^****^P < 0.0001.

**Table 2:** Correlation between THY1 expression and gene markers of dendritic cell in LUSC.

**Cancer type**	**Description**	**Gene markers**	**Tumor**		**Normal**	
			**R**	***P***	**R**	***P***
LUSC	Dendritic cell	HLA-DPB1	0.35	^****^	0.12	0.42
		HLA-DQB1	0.23	^****^	0.084	0.56
		HLA-DRA	0.34	^****^	−0.059	0.69
		HLA-DPA1	0.37	0	0.074	0.61
		BDCA-1 (CD1C)	0.2	^****^	0.1	0.48
		BDCA-4 (NRP1)	0.45	0	0.48	^***^
		CD11c (ITGAX)	0.27	^****^	0.27	0.057

Abbreviation: LUSC: Lung Squamous Cell Carcinoma; ^****^P < 0.0001.

## DISCUSSION

Nowadays, LUCA is still a leading cause of death not only in developing countries but also in developed countries. Approximately 70% of patients were advanced stage or have distant metastases at the time of diagnosis for the atypical early symptoms and lack of specific early screening biomarkers [18]. A variety of cytotoxic drugs (e.g., platinum, taxane), targeted drugs (e.g., gefitinib, erlotinib) and immune checkpoint inhibitors (e.g., PD1/PDL-1 inhibitors) have been used in the treatment of non-small cell lung cancer, and have achieved certain some curative effect [18–22]. However, the overall prognosis of LUCA was un-satisfactory for the inherent or acquired drug resistance. Therefore, it is important to put great efforts to identify more specific biomarkers of LUCA.

Recent years, molecular biology technology and bioinformatics have been widely developed, which provide a new insight to explore the functional mechanism and identify novel biomarkers for human cancers. In our study, we mainly used some online public databases (e.g., ONCOMINE, GEPIA, UALCAN, TIMER) to comprehensively explore the expression level and prognostic significance of *THY1* in LUSC. In summary, we confirmed that *THY1* was significantly up-regulated at the level of mRNA in LUSC. Furthermore, over-expression of *THY1* was significantly correlated with poor survival in (e.g., OS, DFS) in LUSC. Further analysis revealed that CpG islands methylation of *THY1* was negatively correlated with *THY1* mRNA expression in LUSC. We also attempted to explore the molecular mechanism of *THY1* in LUSC. Functional enrichment analysis of correlated genes of *THY1* revealed that it may get involved in the process of ECM organization and EMT, which were reported to play pivotal roles in the occurrence and development of human diseases [23–26]. Studies have proved that the increased expression of key molecules of EMT signaling pathway (e.g., TWIST1, MMPs) was often accompanied by the increase of immune cell infiltration abundance in tumor microenvironment in breast cancer, which contributed to the immune escape of tumor cells [27]. Furthermore, many studies have confirmed that in a variety of cancers, the process of EMT was often accompanied by desensitization of immunotherapy drugs [28–30]. Our study indicated that differentially expressed *THY1* not only got involved in the process of EMT, but also significantly correlated with the immune infiltration levels in LUSC. It provides us with a new therapeutic strategy for LUSC by blocking *THY1* to remolding ECM and tumor microenvironment.

The development of immunotherapy has brought hope for the treatment of lung cancer, especially for advanced or recurrent patients [31, 32]. However, only a small portion of patients can benefit from immunotherapy. The commonly used biomarkers for efficacy prediction in lung cancer include the expression of PD-L1, tumor mutational burden, specific genomic alterations, and circulating tumor DNA [31, 33]. These biomarkers can help predict the response to immune checkpoint inhibitors and other cancer therapies, and may also be used to guide treatment decisions. Therefore, it is crucial to identify more biomarkers that can predict or improve the effectiveness of immunotherapy. In our study, we found that *THY1* was positively correlated with the infiltration levels of certain immunocytes (e.g., B cell, CD8^+^ T cell, CD4^+^ T cell, macrophage, neutrophil and dendritic) in LUSC, which indicates that *THY1* may play pivotal roles in immune microenvironment remodeling. Developing effective inhibitors targeting *THY1* may be beneficial for improving immunotherapy and is worth exploring in the future.

It is no doubt that there are some limitations in our study. Firstly, our results were mainly derived from public databases, which need to be further verified in near future. Cell or animal models of LUSC overexpressing *THY1* would be helpful to further confirm our results. Secondly, we can use the DNA methylation agonists to further verify the relationships between *THY1* CpG islands methylation and mRNA expression. Finally, the mechanism of differentially expressed *THY1*, EMT, and immune infiltration require further experiments to verify in future.

## MATERIALS AND METHODS

### GEPIA analysis

GEPIA (http://gepia.cancer-pku.cn/) contains the TCGA samples, which can provide key interactive information and customized functions, including tumor/normal differential expression profile analysis, profile drawing, pathological staging, patient survival analysis, similar gene detection analysis, etc. In this study, we used the GEPIA to explore the *THY1* expression landscape in human cancers and normal organs [34]. We also used it to explore the prognostic values of *THY1* on 33 types of cancer in TCGA. Finally, we used it to further validate the relationships between *THY1* expression and gene markers.

### TIMER analysis

TIMER (https://cistrome.shinyapps.io/timer/) is an online database, which mainly uses RNA SEQ expression profile data to detect the infiltration of immune cells in tumor tissues. It provides the infiltration of six types of immune cells (B cells, CD4^+^ T cells, CD8^+^ T cells, neutrophils, macrophages, and dendritic cells) [35]. Here, we firstly used it to measure the *THY1* expression levels in 33 types of cancer from TCGA. We then used it and explored the relationships between *THY1* expression and immune lymphocytes infiltration levels. The prognostic values of *THY1* expression and immune lymphocytes, and the relationships between *THY1* expression and immune lymphocytes gene markers were also measured by TIMER.

### ONCOMINE analysis

ONCOMINE (http://www.oncomine.org/resource/login.html) database integrates RNA and DNA-seq data from sources such as GEO, TCGA, and published literature, and contains a wealth of oncogene chips and integrated data to facilitate relevant analysis by researchers [36, 37]. Here, we mainly used it to explore the expression level of *THY1* in many types of cancers.

### PrognoScan analysis

PrognoScan (http://dna00.bio.kyutech.ac.jp/PrognoScan/) integrates large number of microarray data sets with prognostic information. The site basically includes most of the tumor data, which can be used to analyze the relationship between gene expression and patient prognosis, such as OS and DFS [38]. Here, we also used it to measure the prognostic values of differentially expressed *THY1* in many malignant tumors.

### UALCAN analysis

UALCAN (https://ualcan.path.uab.edu/analysis.html) is a website for online analysis and mining of cancer data from the TCGA database. It helps preliminary research on whether relevant genes can be used as biomarkers, expression profiling, survival analysis, etc., [39]. In our study, the UACLAN was mainly used to further explore the relationships between the expression of *THY1* and clinicopathological parameters (individual cancer stage, age, smoking habits, nodal metastasis status, and TP53 mutation status) in LUSC.

### Kapan-Meier plotter analysis

Kaplan-Meier plotter (https://kmplot.com/analysis/) is an online database which can measure the prognostic significance of interesting genes on 21 types of human cancer at the level of mRNA, miRNA, and protein [40]. In this study, we mainly used it to assess the prognostic values of *THY1* expression under restricted clinicopathological parameters in LUSC.

### MEXPRESS analysis

MEXPRESS (https://mexpress.ugent.be/) is an online database containing 33 kinds of cancer multi omics data from TCGA. Users can easily view the relationships between the interested genes expression, DNA methylation and clinical characteristics [41]. Here, we used it to explore the relationships of different CpG islands methylation and *THY1* expression in LUSC.

### TCGA wander analysis

TCGA Wander (http://maplab.imppc.org/wanderer/) is an online database based on TCGA data, and users can use it to intuitively explore the relationship between DNA methylation and gene expression [42]. We used it to explore the mean *THY1* methylation and *THY1* mRNA expression of LUSC and normal lung tissues.

### Gene set cancer analysis (GSCA) analysis

GSCA (https://guolab.wchscu.cn/GSCA/#/) is an online database that contains TCGA data and can perform integrated genomic and immunogenomic analysis. It is a multifunctional database composed of 4 modules: differential expression analysis, immune infiltration analysis, gene mutation analysis and drug screening. Here, we used it to explore the relationships between *THY1* methylation level and mRNA expression in LUSC from TCGA [43].

### LinkedOmics analysis

LinkedOmics (https://linkedomics.org/login.php) is a friendly and easy-to-operate online database which contains 32 types of human cancer multi-omics data from the TCGA database and a database of mass spectrometry-based proteomics data generated by the Clinical Proteomics Tumor Analysis Association (CPTAC) [44]. Here, we used the LinkedOmics to identify positively and negatively correlated genes of *THY1* in LUSC.

### STRING analysis

SRTING (https://cn.string-db.org/) is an online database for searching known protein interaction relationships. It stores 2031 species, 9,643,763 proteins, and 1,380,838,440 interaction information [45]. We mainly used it to construct the PPI of correlated genes of *THY1* identified by LinkedOmics.

### FunRich3.1.3 analysis

FunRich is an independent software tool, mainly used for gene and protein function enrichment and interaction network analysis. In addition, the analysis results can be graphically described in the form of Venn, bar, column, pie, and donut charts [46]. In this study, we mainly performed enrichment analysis of *THY1* correlated genes to identify potential functional mechanism.

### Availability of data and materials

The data used in our study are available from the ONCOMINE (http://www.oncomine.org/), UALCAN (https://ualcan.path.uab.edu/analysis.html), LinkedOmics (http://www.linkedomics.org/login.php), Gene Expression Profiling Interactive Analysis (http://gepia.cancer-pku.cn/detail.php), Kaplan-Meier plotter (http://www.kmplot.com), TIMER (http://cistrome.shinyapps.io/timer/), STRING (http://string-db.org), PrognoScan (http://dna00.bio.kyutech.ac.jp/PrognoScan/), GSCA (http://bioinfo.life.hust.edu.cn/GSCA/#/), TCGA Wander (http://maplab.imppc.org/wanderer/doc.html), MEXPRESS (https://mexpress.be/).

## Supplementary Materials

Supplementary Figures

Supplementary Table 1
