# Supplementary material for: THY1 is a prognostic-related biomarker via mediating immune infiltration in lung squamous cell carcinoma (LUSC)
Source: Aging (Albany NY). 2024 May 30;16(11):9498–517. doi: 10.18632/aging.205880 (PMC11210259; doi:10.18632/aging.205880)
Supplement: Supplementary Figures [file aging-16-205880-s001.pdf]

SUPPLEMENTARY FIGURES

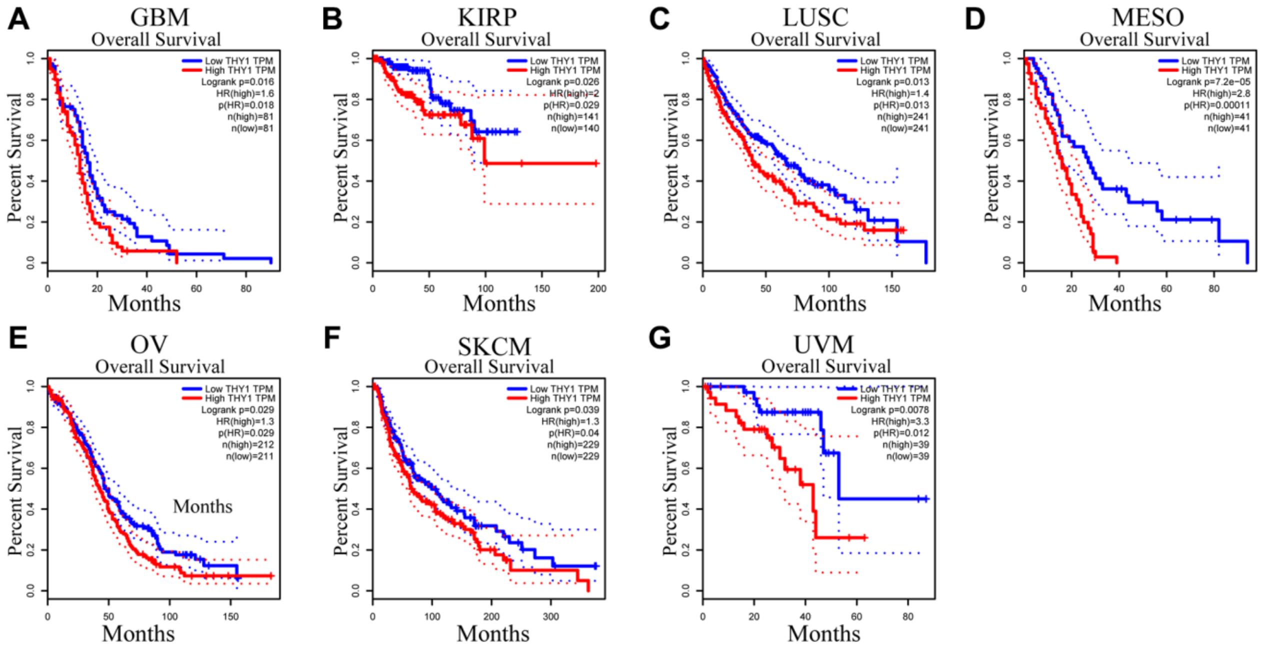

Supplementary Figure 1. The prognostic values (overall survival) of differentially expressed *THY1* in many types of cancers. (**A**–**G**) Kaplan-Meier survival curves of up-regulated *THY1* in glioblastoma multiforme (GBM), kidney renal papillary cell carcinoma (KIRP), lung squamous cell carcinoma (LUSC), mesothelioma (MESO), ovarian cancer (OV), skin cutaneous melanoma (SKCM), and uveal melanoma (UVM) from GEPIA database.

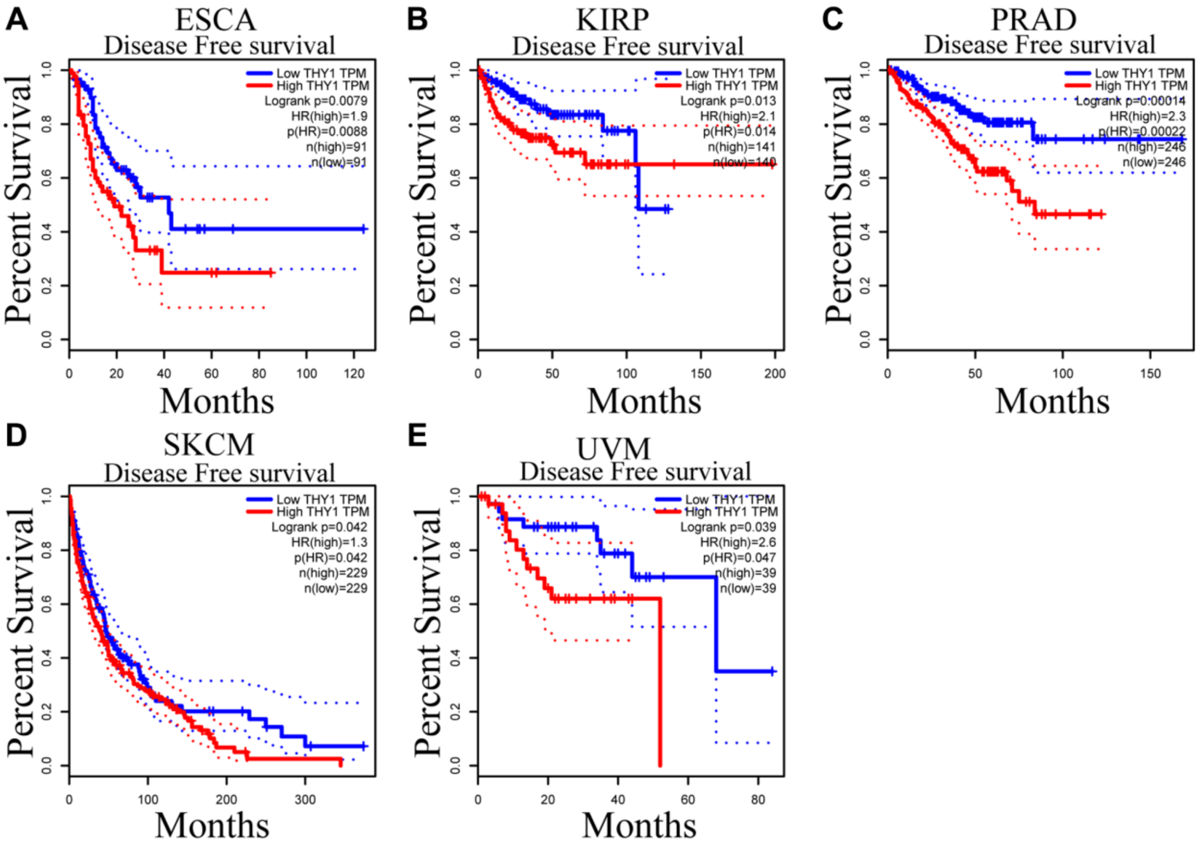

Supplementary Figure 2. The prognostic values (disease free survival) of differentially expressed *THY1* in many types of cancers. (**A**–**E**) Kaplan-Meier survival curves of up-regulated *THY1* in esophageal carcinoma (ESCA), kidney renal papillary cell carcinoma (KIRP), prostate adenocarcinoma (PRAD), skin cutaneous melanoma (SKCM), and uveal melanoma (UVM) from GEPIA database.

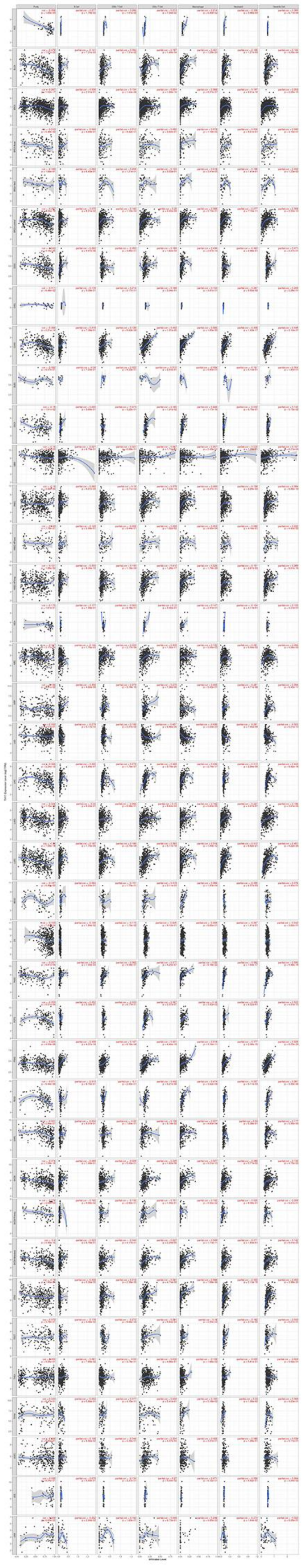

Supplementary Figure 3. The relationships between *THY1* expression and immune infiltration levels in 33 types of cancer form TCGA in TIMER database.
