# Supplementary material for: THY1 is a prognostic-related biomarker via mediating immune infiltration in lung squamous cell carcinoma (LUSC)
Source: Aging (Albany NY). 2024 May 30;16(11):9498–517. doi: 10.18632/aging.205880 (PMC11210259; doi:10.18632/aging.205880)
Supplement: Supplementary Table 1 [file aging-16-205880-s002.pdf]

SUPPLEMENTARY TABLE

Supplementary Table 1. Kaplan-Meier plotter to determine the effect of different clinicopathological factors on the expression of THY1 gene and clinical prognosis in LUSC.

Clinicopathological characteristics	OS			FP			PPS		
	N	**HR**	***P***	***N***	**HR**	***P***	***N***	**HR**	***P***
**Sex**									
Female	129	0.47 (0.26–0.85)	0.011	29	0.03 (0–0.29)	2.60E-06	4	/	/
Male	342	1.3 (0.99–1.72)	0.06	112	0.62 (0.34–1.14)	0.12	16	/	/
**Stage**									
1	172	0.84 (0.54–1.31)	0.44	33	0.29 (0.08–1.1)	0.054	9	/	/
2	100	0.4 (0.2–0.82)	0.0093	24	0.36 (0.07–2.03)	0.23	6	/	/
3	43	1.56 (0.77–3.17)	0.21	9	/	/	5	/	/
**Grade**									
I	22	0.59 (0.19–1.78)	0.34	0	/	/	0	/	/
II	73	0.58 (0.27–1.2)	0.14	0	/	/	0	/	/
III	15	/	/	0	/	/	0	/	/
**AJCC Stage T**									
1	106	1.6 (0.92–2.79)	0.094	9	/	/	3	/	/
2	160	0.61 (0.38–0.99)	0.042	32	0.28 (0.03–2.3)	0.2	7	/	/
3	25	1.9 (0.6–6)	0.27	0	/	/	0	/	/
4	13	/	/	0	/	/	0	/	/
**AJCC Stage *N***									
0	208	1.27 (0.85–1.88)	0.24	25	0.35 (0.04–3.08)	0.33	6	/	/
1	252	0.72 (0.52–0.99)	0.045	130	0.45 (0.28–0.72)	0.00065	71	0.73 (0.43–1.24)	0.24
2	19	/	/	0	/	/	0	/	/
**AJCC Stage M**									
0	300	0.72 (0.49–1.05)	0.085	41	0.4 (0.11–1.44)	0.15	10	/	/
1	10	/	/	0	/	/	0	/	/
**Surgery success only surgical margins negative**	65	1.98 (0.75–5.26)	0.16	0	/	/	0	/	/
**Chemotherapy**									
Yes	42	4.38 (0.76–25.43)	0.079	15	/	/	8	/	/
No	35	2.94 (0.9–9.56)	0.06	10	/	/	2	/	/

Abbreviations: LUSC: Lung Squamous Cell Carcinoma; OS: Overall Survival; FP: First Progression; PPS: Post-progression Survival; HR: Hazard Ratio.
